# Long-Term Effects of Autologous Bone Marrow Stem Cell Treatment in Acute Myocardial Infarction: Factors That May Influence Outcomes

**DOI:** 10.1371/journal.pone.0037373

**Published:** 2012-05-24

**Authors:** David M. Clifford, Sheila A. Fisher, Susan J. Brunskill, Carolyn Doree, Anthony Mathur, Mike J. Clarke, Suzanne M. Watt, Enca Martin-Rendon

**Affiliations:** 1 Stem Cell Research Laboratory, NHS-Blood and Transplant, John Radcliffe Hospital, Oxford, United Kingdom; 2 Nuffield Department of Clinical Laboratory Sciences, University of Oxford, Oxford, United Kingdom; 3 Systematic Review Initiative, Clinical Research Group, NHSBT-Oxford, John Radcliffe Hospital, Oxford, United Kingdom; 4 Department of Clinical Pharmacology, William Harvey Research Institute, London, United Kingdom; 5 All-Ireland Hub For Trials Methodology Research, Queen’s University, Belfast, United Kingdom; 6 Barts and the London NIHR Biomedical Research Unit, London, United Kingdom; Sapienza University of Rome, Italy

## Abstract

**Aims:**

To investigate whether there are important sources of heterogeneity between the findings of different clinical trials which administer autologous stem cell treatment for acute myocardial infarction (AMI) and to evaluate what factors may influence the long-term effects of this treatment.

**Methods and Results:**

MEDLINE (1950-January 2011), EMBASE (1974-January 2011), CENTRAL (*The Cochrane Library* 2011, Issue 1), CINAHL (1982-January 2011), and ongoing trials registers were searched for randomised trials of bone marrow stem cells as treatment for AMI. Hand-searching was used to screen recent, relevant conference proceedings (2005–2010/11). Meta-analyses were conducted using random-effects models and heterogeneity between subgroups was assessed using chi-squared tests. Planned analyses included length of follow-up, timing of cell infusion and dose, patient selection, small trial size effect, methodological quality, loss of follow-up and date of publication. Thirty-three trials with a total of 1,765 participants were included. There was no evidence of bias due to publication or time-lag, methodological quality of included studies, participant drop-out, duration of follow-up or date of the first disclosure of results. However, in long-term follow-ups the treatment seemed more effective when administered at doses greater than 10^8^ cells and to patients with more severe heart dysfunction.

**Conclusions:**

Evaluation of heterogeneity between trials has not identified significant sources of bias in this study. However, clinical differences between trials are likely to exist which should be considered when undertaking future trials.

## Introduction

Although advanced therapies have improved short-term survival following acute myocardial infarction (AMI), the incidence of heart failure is steadily increasing worldwide [1]. Current treatments do not address the substantial loss of tissue through injury nor cell death incurred during AMI [2]. In the last decade, autologous bone marrow stem cell (BMSC) treatment has aimed to complement thrombolytic therapies and primary angioplasty in the treatment of AMI (for review see [3]). The hypothesis has been that BMSC would improve heart function delaying the progression of the disease. There is now a substantial body of evidence from randomised trials to assess the effects of this treatment, and a recent update of a Cochrane review by several of the authors of this paper has systematically reviewed this evidence [4].

The first clinical trials were designed to test the safety and feasibility of this new treatment, but were not necessarily powered to assess its efficacy and long-term effects on survival free of major associated cardiac events [5], [6], [7], [8], [9]. To date the treatment appears safe and associated with low mortality and morbidity rates (for review see [3], [10]). However, there is controversial evidence that a beneficial effect on global heart function is significant and persist long-term (for review see [5], [11] and references therein). Clinical evidence from randomised trials of intracoronary infusion of BMSC post-AMI have been evaluated previously in several meta-analyses [10], [12], [13]. The major limitations in the field that may contribute to these conflicting results among trials include the small trial sizes and differences in patient selection, participants lost to follow-up, cell isolation protocols, cell dose/type, timing of cell infusion, route of delivery and methodologies used to measure surrogates; as well as variation in data acquisition and data analysis protocols. In addition, new interventions generally raise concerns that early optimism is fuelled by extreme results in early disclosure just to be contradicted by later results [14], [15], [16]. The rationale of the underlying Cochrane systematic review was to evaluate the efficacy of any dose of autologous BMSC administered to patients with a diagnosis of AMI following revascularisation [4]. In our previous study, sub-group analyses were planned to assess the effect of using different methods (e.g. magnetic resonance imaging (MRI), echocardiography, left ventricular angiography, single positron emission computed tomography (SPECT) or radionucleide ventriculography (RNV)) to measure heart function [4]. The aim of the present study was to conduct further risk of bias and sub-group analyses to explore whether the overall estimate of treatment effect size is a reliable guide to its effect and therefore to address some of the limitations in the field. Here, pre-planned analyses included (i) small trial size effect, (ii) trial quality and participants lost to follow-up, (iii) length of follow-up in the trial design, (iv) date of publication bias, (v) timing of cell infusion, (vi) cell dose/type, (vii) route of delivery and (viii) differences in patient selection.

## Methods

### Eligibility

Inclusion criteria: (i) randomized trials, (ii) participants with a clinical diagnosis of AMI, (iii) within a month of receiving re-vascularisation by percutaneous coronary intervention (PCI) or thrombolytic therapy or both, (iv) any dose of autologous BMSC, (v) any route of administration, (vi) in the comparator arm participants did not receive BMSC and (vii) any co-interventions provided they were equally applied to each trial arm.

### Search Strategy

The search strategy is detailed elsewhere [4]. Briefly, databases were searched through to January 31^st^ 2011 for randomised trials in which BMSC were administered as treatment for AMI, including MEDLINE (1950–2011), EMBASE (1974–2011), CENTRAL (*The Cochrane Library* 2011, Issue 1), CINAHL (1982–2011), PubMed, Lilacs, and the Transfusion Evidence Library. Ongoing trial registers (ClinicalTrials.gov, the ISRCTN Register and the WHO International Clinical Trials Platform Registry) were also searched. Searches were combined with adaptations of the Cochrane highly sensitive RCT search filter in MEDLINE, EMBASE and CINAHL [17]. No restrictions by language, year of publication or publication status were imposed. Proceedings from the American Heart Association (2005–2010) and International Society of Stem Cell Research (2005–2011) conferences and the reference lists of identified studies and relevant review articles were hand searched for additional studies.

### Data Extraction

For each eligible trial, the study and patient population characteristics, the nature of the intervention and comparator, and the outcomes assessed were extracted. The quality of the studies was assessed on the bases of generation of random sequence, concealment of treatment allocation, blinding of outcome assessment and adequacy of follow-up [18]. Eligibility screening, data extraction and assessment of methodological quality were undertaken independently by a total of three reviewers, such that at least two reviewers looked at each potentially eligible trial. Where a trial had used several methods for outcome assessment (e.g. echocardiography, MRI, SPECT, RNV or left ventricular angiography), MRI data were preferentially included in our analysis.

### Statistical Analysis

Outcome data were analysed quantitatively using RevMan 5 and presented as relative risk (RR) for dichotomous outcomes or weighted mean difference (WMD) for continuous outcomes with 95% confidence interval (CI), two-sided significance tests are reported. Meta-analyses were undertaken using random effects models, due to the high degree of heterogeneity present in these studies [4], [10]. Statistical heterogeneity was examined using the I^2^ statistic [19] and the chi-squared test.

### Sensitivity Analysis

Bias related to study size (such as publication bias [20]) was assessed by Funnel plots with Egger’s test used to assess asymmetry. Sensitivity analyses were undertaken for all relevant included data to assess the influence of (i) the methodological quality of the trials, (ii) the length of follow-up, (iii) participant drop-out and (iv) publication date. These analyses were specified before they were conducted. In the first instance, trials where the generation of random sequence was rated adequate (marked YES) were analysed separately from those where the generation of random sequence was rated as unclear (marked UNCLEAR) or inadequate (marked NO) as a possible explanation for observed statistical heterogeneity. To assess the effect of length of follow-up on results, trials with short term follow-up periods that have been followed up long term were analyzed separately from those with no long-term follow-up. The influence of participants drop-out was determined by analyzing separately trials with less than 20% drop-out, randomised trials with 20 to 50% drop-out and randomised trials with greater than 50% drop-out for major outcomes measured as dichotomous data (e.g. mortality, reinfarction and target vessel revascularisation). Finally, trials were sub-grouped on the basis of their by start date, end date, publication of the main/full article and disclosure or publication of the first results on the primary outcome (LVEF), to assess the possibility of a relationship between publication date and effect size. Other potential reasons for observed heterogeneity were explored via sub-group analysis, with particular emphasis placed on clinical, treatment and outcome measurement differences among the included studies. Sub-groups were stratified by the timing of BMSC transplantation from onset of AMI, dose of BMSC administered, route of administration and baseline LVEF. Differences in effect size between subgroups were assessed using chi-squared tests for heterogeneity between sub-groups as implemented in RevMan 5.

## Results

### Description of the Included Studies and Summary of Previous Findings

The search strategy followed has been described in detail elsewhere [4]. A total of 2,169 citations were identified in the initial search of which thirty-three were primary references to eligible studies (Figure S1). The characteristics of all included studies are detailed in Tables S1 and S5. The thirty-three included randomised trials represent thirty-nine treatment comparisons where BMSC was compared with control in 1,765 patients. Treatment comparisons were defined following the criteria previously described (for review see also [4]). Clinical outcomes and efficacy of BMSC treatment following AMI are fully described in detail in our previous study [4] and are summarised here and in supplementary material for clarity. BMSC administration within a month of AMI has no significant effect on mortality, morbidity, or adverse events. Cumulative figures are presented as ≤61 months follow-up in supplementary data (Table S2 and reference to studies in Table S5). The statistical heterogeneity in this case was negligible (I^2^ = 0–11%). However, a statistical power calculation showed that a study with over 5,000 participants in each randomised group would be required in order to achieve 80% power to detect any significant difference in mortality rates given the relatively low incidence of death within the follow-up period for these trials (2.8% in BMSC and 3.6% in Control groups, respectively). BMSC treatment significantly improved left ventricular volumes and ejection fraction in short- and long-term follow-up periods. However, a considerable degree of statistical heterogeneity (I^2^>75%) was observed in both infarct size and LVEF comparisons. A summary is presented in supplementary data (Table S3 and reference to studies Table S5). As global LVEF and infarct size have been used as surrogates in many of the included trials, this study was design to explore the observed heterogeneity using these two outcome measures.

### Exploring Heterogeneity: Risk of Bias

In order to explore the heterogeneity observed above and to dispel concerns raised with novel interventions, risks of bias were assessed according to the criteria described in the Methods.

**Figure 1:**
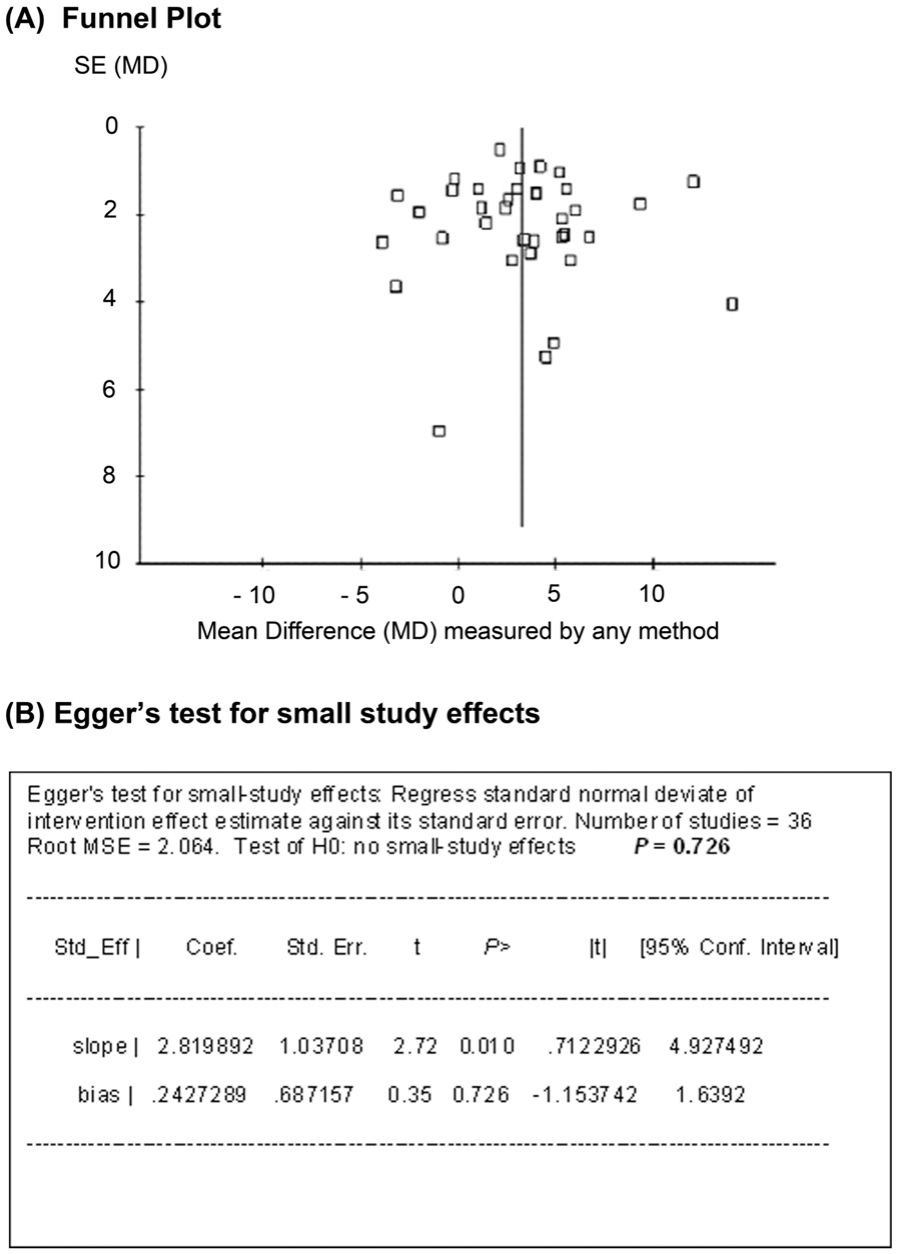
Assessment of risk of bias due to publication and study size on LVEF. (A) Funnel plot and (B) Egger’s test. No significant risk of publication bias or small study effects was observed.

**Figure 2:**
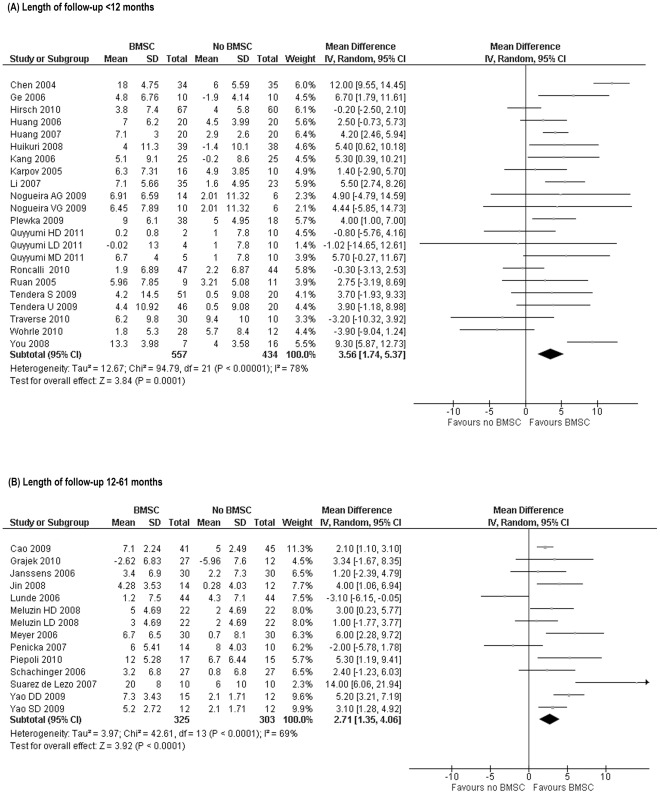
Forest plot of Weighted Mean Difference [WMD, with 95% CI (confidence interval)] in left ventricular ejection fraction (LVEF) in short-term follow-up. (A) Twenty-two randomised trials reporting only short-term follow-up and (B) the remaining 14 trials that reported long-term outcome data as well as short-term data. BMSC treatment significantly improved LVEF in trials with short-term follow-up (3.56%, 95% CI 1.74 to 5.37, p<0.0001) as well as in trials with short- and long-term follow-up (2.71%, 95% CI 1.35 to 4.06, p<0.0001).

**Figure 3:**
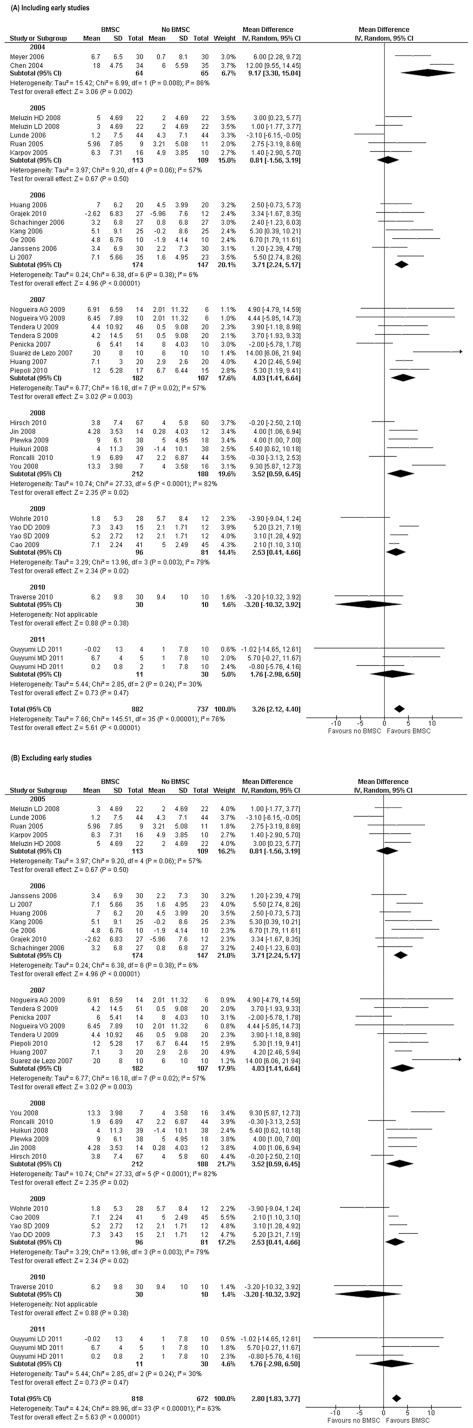
Forest plot of Weighted Mean Difference [WMD, with 95% CI (confidence interval)] in left ventricular ejection fraction (LVEF) in short-term follow-up sub-grouped by the year of the first results’ disclosure. (A) Including early studies reporting data in 2004 (3.26%, 95% CI 2.12 to 4.40, p<0.00001).and (B) excluding studies with early reporting in 2004 (2.80%, 95% CI 1.83 to 3.77, p<0.00001). BMSC treatment significantly improved LVEF in both meta-analyses. WMD had overlapping CI and were not significantly different.

- **Risk of publication bias and small study effect.** The possibility of publication bias and study size was assessed by Funnel plot and the Egger’s test (Figure 1A&B). The analysis showed no significant small study effects (p = 0.726) and, therefore, little evidence of publication bias.
- **Methodological quality of included studies.** The quality assessment of the included studies is summarized in supplementary data (Table S4). Sensitivity analysis to estimate the effect of randomization on LVEF was not required as all included studies followed an adequate method of sequence generation during randomization (Table S4) and this could be assessed because of the high quality of reporting of these relatively recent trials.
- **Loss of follow-up bias on mortality and morbidity.** In 37 trials, 80% or more (ranging from 80%–100%) of randomized participants were analyzed by their randomized treatment group. One trial did not report loss of follow-up [21]. In the remaining trial, only 63.64% of randomized participants were included in the analysis [22]. Sensitivity analyses excluding this trial from the meta-analysis [22] had a negligible effect on the effects on mortality during short term (RR 0.80, 95% CI 0.40 to 1.61, p = 0.52 compared to RR 0.75, 95% CI 0.39 to 1.46, p = 0.40) and long term (RR 0.63, 95% CI 0.20 to 2.00, p = 0.43 compared to RR 0.59, 95% CI 0.22 to 1.56, p = 0.29) follow-up. Similar results were observed for incidence of re-infarction, restenosis, hospital readmission and target vessel revascularization, suggesting a negligible risk of bias. Here the original analyses with all included studies are presented (Table S2).
- **Length of follow-up bias on LVEF.** For this purpose, the 36 trials that reported short-term LVEF data were divided into two groups: 22 trials were followed-up only for <12 months (Figure 2A) whilst the remaining 14 trials were followed up for 12–61 months (Figure 2B). BMSC treatment effect on LVEF in trials followed up for <12 months (WMD 3.56%, 95% CI 1.74 to 5.37, p = 0.0001- Figure 2A) was not statistically different from the treatment effect in trials followed-up for 12–61 months (WMD 2.71%, 95% CI 1.35 to 4.06, p<0.0001- Figure 2B). These results suggest that those trials with long-term follow-up are representative of all included studies (Table S1).
- **Disclosure or publication date bias on LVEF.** The influence of study start date or end date and main publication date on the primary outcome (LVEF) was estimated by sorting the included studies according to those dates. Interestingly, no significant effect was observed in any of these comparisons, indicating a negligible risk of bias on treatment effect. However, when trials were grouped by the year when the first results were disclosed, the studies that disclosed their results first (in 2004) [6], [23] showed an average greater effect on LVEF in favour of the treatment than the studies that were designed or reported later (Figure 3A). If we exclude these early trials from the meta-analysis, the overall estimate for the effect on LVEF was reduced (WMD 2.80%, 95% CI 1.83 to 3.77, p<0.001- Figure 3B) compared to the pool of all included studies (WMD 3.26%, 95% CI 2.12 to 4.40, p<0.001- Figure 3A). However, the difference was not substantial and the inclusion of trials with the early, most promising results showed low risk of bias.

### Exploring Heterogeneity: Sub-group Analysis

Planned sub-group analyses were carried out to assess the impact of (i) the timing of the BMSC transplantation following AMI, (ii) the dose of BMSC administered, (iii) the route of administration and (vi) the baseline LVEF on infarct size and LVEF for long term follow-up (Table 1). Timing of administration of BMSC infusion was sub-grouped into ≤7days and >7days, to reflect the median delay to BMSC infusion from AMI across included studies. Our previous work suggested that doses of BMSC >10^8^ would be required to observe a significant change in LVEF in the treated arm compared with the control arm [10]. Therefore, for the purpose of this study, trials were divided into two groups according to dose: ≤10^8^ BMSC and >10^8^ BMSC. All included trials where the route of administration is detailed in their methods administered BMSC via the infarct related coronary artery. Only one trial compared venus and arterial delivery of BMSC [24]. Therefore, the pre-planned analysis subgrouping the trials by route of delivery was deemed not appropriate in the present study. Finally, further analyses were carried out dividing the trials in two groups to reflect the median value of baseline LVEF in the included trials: ≤40% and >40% baseline LVEF.

**Table 1:** Long term follow up (≥12–61months) for infarct size and LVEF: sub-group analysis.

Subgroup Analysis	Subcategory	No. studies(No. participants)	Infarct size (%)WMD (95% CI) *p* value	Ref. to study	No studies(No. participants)	L|VEF (%) WMD (95% CI) *p* value	Ref. to study
**Timing of SCT** a	≤7 days	3 (99)	−5.16(−8.87, −1.44) *P* = 0.007	[9], [45]	6 (241)	4.76 (2.18, 7.33) *P*<0.001	[7], [9], [23], [30], [45], [47]
	>7 days	0 (0)	N/A		3 (56)	5.87 (1.24, 10.51) *P* = 0.01	
	Difference in subcategories					*P* = 0.68	
**Dose**	≤10^8^	3 (168)	−1.96 (−5.23, 1.32) *P* = 0.24	[8], [44]	6 (228)	2.66 (–0.07, 5.38) *P* = 0.06	[8], [22], [44], [45], [48]
	>10^8^	4 (185)	−4.28 (−7.29, −1.27) *P* = 0.005	[9], [45], [47]	9 (407)	4.72 (2.68, 6.76) *P*<0.001	[7], [9], [23], [28], [30], [45], [47], [49], [50]
	Difference in subcategoris		*P* = 0.31			*P* = 0.23	
**Baseline LVEF** b	≤40%	3 (91)	−5.12 (−8.76, −1.48) *P* = 0.006	[44], [45]	7 (177)	5.62 (2.95, 8.29) *P*<0.001	[22], [30], [44], [45], [49], [50]
	>40%	3 (222)	−1.37 (−3.62, 0.88) *P* = 0.23	[8], [9], [47]	7 (418)	2.36 (0.67, 4.04) *P* = 0.006	[7], [8], [9], [23], [28], [44], [47], [48]
	Difference in subcategories		***P*** ** = 0.09**			***P*** ** = 0.04**	

CI, confidence interval; LVEF, left ventricular ejection fraction; N/A, not applicable; SCT, stem cell transplantation; WMD, weighted mean difference.

a studies classified according to timing of stem cell administration in at least 70% of patients (as estimated from the mean and standard error or median and interquartile range).

b Mean%, Melzuin LD was excluded as treatment and control groups in this study could not be classified into the same baseline LVEF group.

- **Timing of BMSC infusion.** Table 1 shows statistically significant changes in both infarct size (WMD = −5.2%, p = 0.007) and LVEF (WMD = 4.8%, p = 0.0003), in favour of BMSC, when the treatment was administered within 7 days post-AMI. At present, there are no data available to assess the long term effect on BMSC on infarct size for treatment administered after 7 days, however a significant difference in LVEF in favour of BMSC was maintained when the treatment was administered later than 7 days (WMD = 5.9%, p = 0.01). No significant differences between subgroups were observed for long-term follow-up LVEF (p = 0.68).
- **BMSC dose.** BMSC treatment had a significant effect on infarct size (WMD = −4.3%, p = 0.005) and LVEF (WMD = 4.7%, p = 0.0001) after long-term follow-up when doses >10^8^ BMSC were administered. In contrast, the infarct size and LVEF showed no significant improvement when doses ≤10^8^ BMSC were administered.
- **Baseline LVEF.** Furthermore, the long-term reduction in infarct size in favour of BMSC treatment was statistically significant when the treatment was administered to participants with baseline LVEF ≤40% (WMD = −5.1%, p = 0.006) whereas no significant effect was observed in participants with baseline LVEF >40% (WMD = −1.4%, p = 0.23). The difference in effect sizes these between subgroups was marginally significant (p = 0.08). The effect of BMSC on LVEF was also greater and more statistically significant when participants had LVEF ≤40% at baseline (WMD = 5.6%, p<0.0001) compared with participants with baseline LVEF >40% (WMD = 2.4%, p = 0.006), the difference in effect size between these subgroups was clearly significant for this outcome (p = 0.04).

Taken together, these data indicate that the timing of BMSC transplantation following AMI, the dose of BMSC administered and the baseline LVEF are factors that may contribute to the clinical heterogeneity observed among the studies included in this meta-analysis.

## Discussion

The present study was designed: (i) to assess potential risks of bias and diversity amongst different randomised trials to address major limitations in the field and (ii) to evaluate what factors may influence the long-term effect of BMSC treatment. This meta-analysis confirms the findings of our previous study [4] that BMSC treatment moderately improves heart function and has as yet not been associated with any significant safety concerns, but does not decrease mortality or morbidity significantly in long-term follow-up (with the caveat that there have been no studies designed to address mortality). Our power calculation estimates that a trial with several thousand participants would be needed to detect significant differences in mortality between treatment and control groups. Recently, the first trial to address mortality associated with BMSC treatment in patients who suffered AMI has been designed and funded by the European Union (EU FP7– BAMI). The BAMI trial will be administering unfractionated bone marrow mononuclear cells to patients who have suffered from AMI, similarly to the majority of trials included in the present study. The results of this trial should allow us to directly answer the question of whether BMSC therapy following AMI can change the prognosis of this disease.

As the results of the present study are not always consistent with those of large randomised trials [7], [8], [25], [26], [27], we have conducted extensive sensitivity analyses to evaluate potential factors that may account for the discrepancies observed. Major limitations in the field have been enumerated earlier in the Introduction. Importantly, the underlying Cochrane systematic review for this study is unique and superior to others previously reported in a number of ways and has allowed us to address some of those issues. Firstly, it is based on a comprehensive search strategy and a protocol approved by the Cochrane Collaboration prior to starting the search. Although the majority of randomised trials were identified through searching the main databases (MEDLINE, EMBASE, CENTRAL and CINAHL), hand-searching identified several crucial references used to assess risk of bias that would have otherwise been missed [7], [21], [26], [28], [29], [30], [31]. Secondly, the robustness of the original systematic review has been tested extensively within this study through a comprehensive sensitivity analysis and risk of bias assessment to explore the presence and impact of heterogeneity. Finally, the large number of trials included here allows particularly powerful assessments of heterogeneity through planned sub-group analysis.

It has been suggested that measurement of surrogates such as LVEF by different methodology may be a risk of bias because of the known limitations of some methods [32]. Analysis of the included studies sub-grouped by the methodology used to measure surrogate outcomes has been addressed elsewhere [4]. It has also been suggested that stopping or reporting early results in randomised trial may affect the perception that the public has of the treatment efficacy in novel interventions [33]. As early results are more limited than the results of a final analysis, concerns have been raised as treatment effects seen early may either not be real or may be overly optimistic. In this study, we have had a unique opportunity to assess whether factors such as short versus long follow-up or early reporting of results may contribute to bias amongst the pooled results of all included trials. The risk of bias due to length of follow-up for LVEF was negligible indicating that those trials with long term follow-up data are representative of all included studies. This conclusion may have implications interpreting early results from trials with long-term follow-up or trials with short term follow-up only. In addition, low risk of bias was observed when studies were grouped by study start date, study end date or publication of main reference to the studies. There were no significant differences of treatment effect on LVEF when two early studies [5], [6] where either included or excluded from the meta-analysis and negligible risk of bias due to quality of included studies or loss of participant’s follow-up. This may also imply that trial design and methodology has not changed drastically amongst trials in the last decade for significant differences to be observed. Although no significant risk of bias was observed in the present study, one cannot exclude the possibility that discrepancies between the different studies may be explained by variability in factors such as cell isolation, data acquisition or data analysis protocols amongst others. Hence the importance of agreeing to standardised protocols in the future. Part of the remit of the BAMI trial (EU-BAMI mentioned above) is to consider the methodologies used to date and produce a standardised technique for bone marrow processing and delivery.

Here, a parallel significant improvement on LVEF and reduction of infarct size was observed. Although caution is advisable in interpreting results from surrogate outcomes, the moderate improvement in LVEF over short- (3.26%) and long-term (3.91%) follow-up is similar to that obtained in previous trials where AMI patients were treated with a combination of thrombolytic therapy and PCI [34], [35]. In the CADILLAC trial, improvement of LVEF correlated with better long-term survival rate [35], [36]. The results of the CADILLAC trial were also consistent with those of the Netherland’s trial, where thrombolytic therapy was administered to patients suffering from AMI [37]. Consequently, the moderate but significant improvement in LVEF in favour of BMSC treatment reported in the present study could be clinically very relevant providing that limitations such as study size could be overcome. Improvement in long-term survival has been suggested by the results of two recent randomised trials [28], [38]. When sub-group analyses were conducted, greater effects on infarct size were observed when BMSC were administered earlier (≤7days). Effects on both infarct size and LVEF were greater when BMSC were administered at doses >10^8^ and to patients with larger infarcts or lower baseline LVEF. This is in agreement with previous published results [10], [39]. Administering cells earlier may reduce infarct size and reduce damage during ventricular remodelling thus preventing or delaying the onset of heart failure. The requirement for a larger dose of BMSC to reduce infarct size and improve LVEF can be explained by the low rates of cell retention in the heart after BMSC infusion [29], [40]. This supports the idea that the treatment may have a paracrine effect [41]. A number of randomised trials are currently addressing the effects of timing of stem cell transplantation [42], [43], cell dose [44], [45], [46], cell delivery [24] and cell composition [27] on global left ventricular function. Although global LVEF has been a primary surrogate measured in the majority of included trials, the results presented here should be carefully considered, as there is still no evidence of clinical efficacy.

In summary, we have addressed some of the limitations present in the field here and elsewhere [4]. However, other limitations such as small study sizes, patient-related factors and variability in protocols still remain. This study shows that risk of bias due to publication, quality of the studies, loss of follow-up, duration of follow-up and date of disclosure of early results is minor among randomised trials that administer BMSC as treatment for AMI. BMSC treatment significantly reduces infarct size and improves LVEF long-term. Factors such as timing of BMSC transplantation, cell dose and baseline LVEF could affect the successful outcome of this treatment. An attempt has now been made with the design of the BAMI trial to standardise the techniques of BMNC isolation and delivery to man and to measure clinically significant end-points such as mortality.

## Supporting Information

Figure S1 **PRISMA diagram.**
(DOC)

Table S1 **Characteristics of the included studies.**
(DOC)

Table S2 **Relative risk of dichotomous clinical outcomes.**
(DOC)

Table S3 **Weighted mean differences of continuous outcomes measured.**
(DOC)

Table S4 **Quality assessment of included studies.**
(DOC)

Table S5 **Reference to studies.**
(DOC)
