# Supplementary figures and images for: Long-Term Effects of Autologous Bone Marrow Stem Cell Treatment in Acute Myocardial Infarction: Factors That May Influence Outcomes
Source: PLoS One. 2012 May 24;7(5):e37373. doi: 10.1371/journal.pone.0037373 (PMC3360027; doi:10.1371/journal.pone.0037373)

**
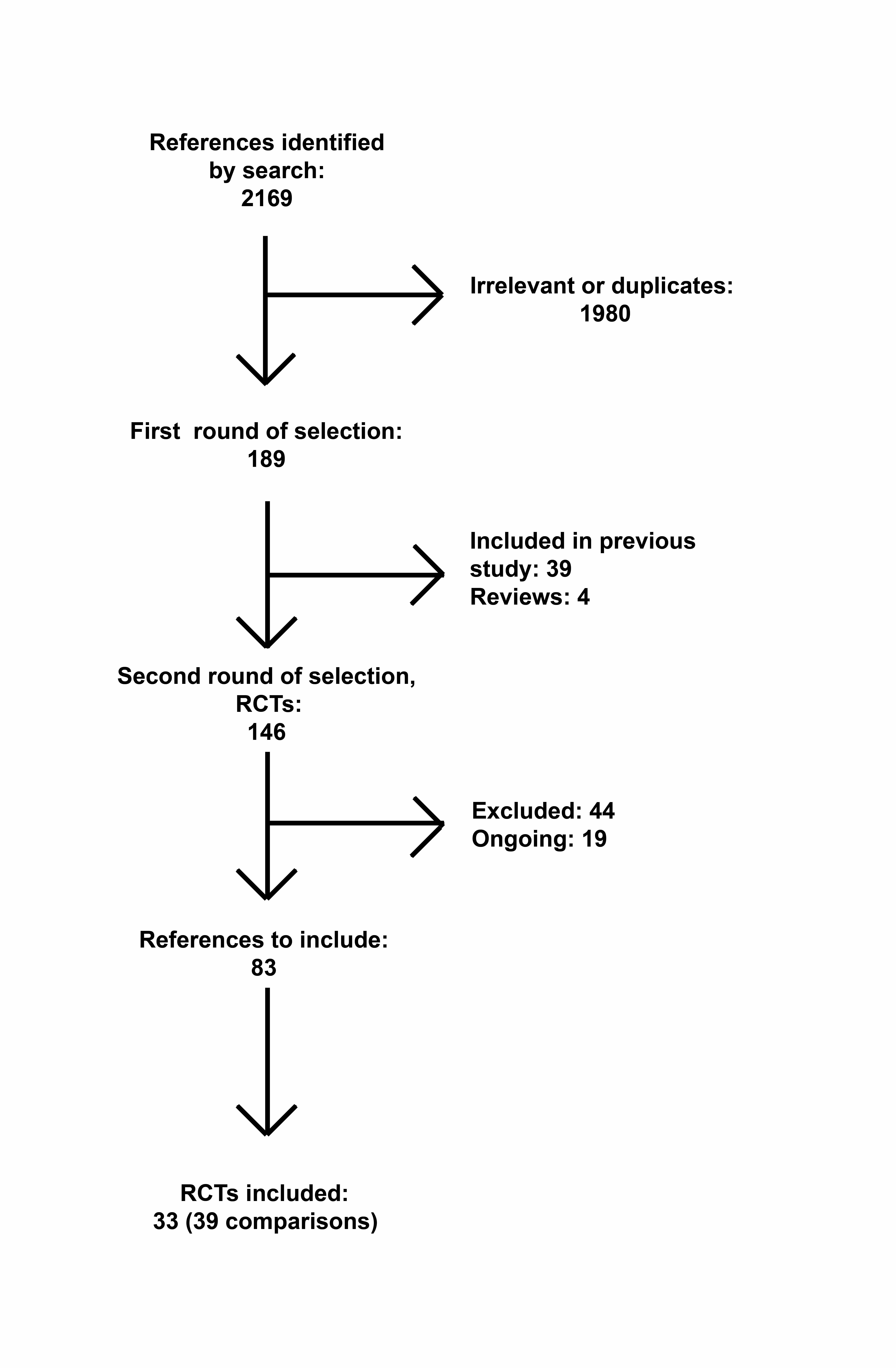
**

Supplement: Figure S1 — PRISMA diagram. (DOC) [file pone.0037373.s001.doc]
