# Supplementary material for: Long-Term Effects of Autologous Bone Marrow Stem Cell Treatment in Acute Myocardial Infarction: Factors That May Influence Outcomes
Source: PLoS One. 2012 May 24;7(5):e37373. doi: 10.1371/journal.pone.0037373 (PMC3360027; doi:10.1371/journal.pone.0037373)
Supplement: Table S2 — Relative risk of dichotomous clinical outcomes. (DOC) [file pone.0037373.s003.doc]

**Table S2:** Relative risk of dichotomous clinical outcomes.

| **Outcome** | **Follow-up** | **No. of trials** | **No. of participants** | | **No. of events** | | **Relative risk (95% CI)** | ***P*-value** | **Reference to studies** |
| --- | --- | --- | --- | --- | --- | --- | --- | --- | --- |
|  |  |  | **BMSC** | **Control** | **BMSC** | **Control** |  |  |  |
| **Mortality** | ≤ 61 mo | 21 | 821 | 681 | 23 | 25 | 0.70 (0.40-1.21) | 0.20 |  |
| **Morbidity**  Reinfarction  Restenosis  Re-admission  TVR | ≤ 61 mo  ≤ 61 mo  ≤ 61 mo  ≤ 61 mo | 20  14  13  11 | 807  484  527  552 | 648  426  492  432 | 16  45  33  72 | 25  43  40  70 | 0.63 (0.35-1.16)  0.90 (0.61-1.31)  0.84 (0.54-1.31)  0.80 (0.59-1.08) | 0.14  0.57  0.44  0.14 |  |
| **Adverse Events** | NR* | 13 | 500 | 426 | 76 | 53 | NA | NA |  |

BMSC = bone marrow stem cells, CI = confidence interval, mo = months, NA = not applicable, NR*= not reported in all trials, TVR = target vessel revascularisation.
