# Supplementary material for: Long-Term Effects of Autologous Bone Marrow Stem Cell Treatment in Acute Myocardial Infarction: Factors That May Influence Outcomes
Source: PLoS One. 2012 May 24;7(5):e37373. doi: 10.1371/journal.pone.0037373 (PMC3360027; doi:10.1371/journal.pone.0037373)
Supplement: Table S3 — Weighted mean differences of continuous outcomes measured. (DOC) [file pone.0037373.s004.doc]

**Table S3**: Weighted mean differences of continuous outcomes measured.

| **Outcome** | **Follow-up** | **No. of trials**  **(No. of participants)** | **WMD (95% CI)** | **P- value** | **I^2^** | **Reference to studies** |
| --- | --- | --- | --- | --- | --- | --- |
| **LVESV** | <12 mo  12-61 mo | 23 (936)  11 (479) | -4.79% (-7.12; -2.47)  -7.36% (-10.61; -4.10) | < 0.0001  < 0.0001 | 59%  77% |  |
| **LVEDV** | <12 mo  12-61 mo | 24 (978)  10 (447) | -2.80 (-5.32; -0.28)  -4.66 ( -7.65; -1.67) | 0.03  0.002 | 55%  52% |  |
| **WMS** | <12 mo  12-61 mo | 10 (564)  4 (279) | -0.07 (-0.17; 0.07)  -0.12 (-0.20; -0.04) | 0.14  0.004 | 88%  67% |  |
| **Infarct size** | <12 mo  12-61 mo | 14 (570)  7 (353) | -1.05 (-2.76; 0.65)  -3.36 (-5.50; -1.22) | 0.23  0.002 | 77%  79% |  |
| **LVEF** | <12 mo  12-61 mo | 36 (1,619)  15 (635) | 3.26 (2.12; 4.40)  3.91 (2.33; 5.49) | <0.00001  <0.00001 | 76%  77% |  |

**Table S3**: Weighted mean differences of continuous outcomes measured (continued).

Footnote:

CI, confidence interval; I^2^, I-squared statistics; LVEDV, left ventricular end-diastolic volume; LVEF, left ventricular ejection fraction; LVESV, left ventricular end-systolic volume; mo, months; WMD, weighted mean difference; WMS, wall motion score.
