# Supplementary material for: Long-Term Effects of Autologous Bone Marrow Stem Cell Treatment in Acute Myocardial Infarction: Factors That May Influence Outcomes
Source: PLoS One. 2012 May 24;7(5):e37373. doi: 10.1371/journal.pone.0037373 (PMC3360027; doi:10.1371/journal.pone.0037373)
Supplement: Table S4 — Quality assessment of included studies. (DOC) [file pone.0037373.s005.doc]

**Table S4:** Quality assessment of included studies.

| **Trial** | **Randomisation sequence generation** | **Allocation concealment** | **Blinding of outcome assessors** | **Loss of follow-up (%)** | **All patients treated in their assigned groups?** |
| --- | --- | --- | --- | --- | --- |
| Cao 2009 | Y | Y | Y | 0 | Y |
| Chen 2004 | Y | Y | Y | 0 | Y |
| Fernandez-Pereira 2006 | Y | Y | Y | U | U |
| Ge 2006 | Y | Y | Y | 0 | Y |
| Grajek 2010 | Y | Y | Y | 0 | Y |
| Hirsch 2010 | Y | N | N | 0 | Y |
| Huang 2006 | Y | Y | Y | 0 | Y |
| Huang 2007 | Y | Y | Y | 0 | Y |
| Huikuri 2008 | Y | Y | Y | 0 | Y |
| Janssens 2006 | Y | Y | Y | 0 | Y |
| Jin 2008 | Y | Y | Y | 0 | Y |
| Kang 2006 | Y | Y | N | 10.7 | Y |
| Karpov 2005 | Y | Y | N | 0 | Y |
| Li 2007 | Y | Y | Y | 17.1 | Y |
| Lunde 2006 | Y | Y | Y | 0.99 | Y |
| Meluzin LD 2008 | Y | Y | Y | 0 | Y |

**Table S4:** Quality assessment of included studies (continued).

| **Trial** | **Randomisation sequence generation** | | **Allocation concealment** | | **Blinding of outcome assessors** | | **Loss of follow-up (%)** | | **All patients treated in their assigned groups?** | |  |
| --- | --- | --- | --- | --- | --- | --- | --- | --- | --- | --- | --- |
| Meluzin HD 2008 | | Y | | Y | | Y | | 0 | | Y | |
| Meyer 2006 | | Y | | Y | | Y | | 7.7 | | Y | |
| Nogueira AG 2009 | | Y | | Y | | Y | | 0 | | Y | |
| Nogueria VG 2009 | | Y | | Y | | Y | | 0 | | Y | |
| Penicka 2007 | | Y | | Y | | Y | | 0 | | Y | |
| Piepoli 2010 | | Y | | Y | | Y | | 0 | | Y | |
| Plewka 2009 | | Y | | Y | | Y | | 0 | | Y | |
| Quyyumi LD 2011 | | Y | | Y | | Y | | 0 | | Y | |
| Quyyumi MD 2011 | | Y | | Y | | Y | | 0 | | Y | |
| Quyyumi HD 2011 | | Y | | Y | | Y | | 0 | | Y | |
| Roncalli 2010 | | Y | | Y | | Y | | 8.9 | | Y | |
| Ruan 2005 | | Y | | Y | | Y | | 0 | | Y | |
| Schachinger 2006 | | Y | | Y | | Y | | 0 | | Y | |
| Suarez de Lezo 2007 | | Y | | Y | | Y | | 0 | | Y | |

**Table S4:** Quality assessment of included studies (continued).

| **Trial** | **Randomisation sequence generation** | | | **Allocation concealment** | | **Blinding of outcome assessors** | | **Loss of follow-up (%)** | | **All patients treated in their assigned groups?** |
| --- | --- | --- | --- | --- | --- | --- | --- | --- | --- | --- |
| Tendera S 2009 | | Y | Y | | Y | | 0 | | Y | |
| Tendera U 2009 | | Y | Y | | Y | | 0 | | Y | |
| Traverse 2010 | | Y | Y | | Y | | 0 | | Y | |
| Wohrle 2010 | | Y | Y | | Y | | 0 | | Y | |
| Yao 2006 | | Y | N | | Y | | 5.4 | | Y | |
| Yao DD 2009 | | Y | Y | | Y | | 10 | | Y | |
| Yao SD 2009 | | Y | Y | | Y | | 20 | | Y | |
| You 2008 | | Y | Y | | N | | 0 | | Y | |
| Zhukova 2009 | | Y | Y | | Y | | 36.4 | | Y | |

N = no, U = unclear or not reported, Y =yes.
