# Supplementary material for: Long-Term Effects of Autologous Bone Marrow Stem Cell Treatment in Acute Myocardial Infarction: Factors That May Influence Outcomes
Source: PLoS One. 2012 May 24;7(5):e37373. doi: 10.1371/journal.pone.0037373 (PMC3360027; doi:10.1371/journal.pone.0037373)
Supplement: Table S5 — Reference to studies. (DOC) [file pone.0037373.s006.doc]

**Table S5: Reference to studies**

1. Cao F, Sun D, Li C, Narsinh K, Zhao L, et al. (2009) Long-term myocardial functional improvement after autologous bone marrow mononuclear cells transplantation in patients with ST-segment elevation myocardial infarction: 4 years follow-up. Eur Heart J 30: 1986-1994.

2. Chen SL, Fang WW, Ye F, Liu YH, Qian J, et al. (2004) Effect on left ventricular function of intracoronary transplantation of autologous bone marrow mesenchymal stem cell in patients with acute myocardial infarction. Am J Cardiol 94: 92-95.

3. Fernandez-Pereira C, Vigo C, Bataglia S, De la Hoz R, Vetulli H, et al. (2006) Autologous bone marrow stem cell transplant after myocardial infarction. In-hospital and long-term follow-up results of the randomised argentina trial (STAR AMI). Eur Heart J August: 279.

4. Ge J, Li Y, Qian J, Shi J, Wang Q, et al. (2006) Efficacy of emergent transcatheter transplantation of stem cells for treatment of acute myocardial infarction (TCT-STAMI). Heart 92: 1764-1767.

5. Grajek S, Popiel M, Gil L, Breborowicz P, Lesiak M, et al. (2010) Influence of bone marrow stem cells on left ventricle perfusion and ejection fraction in patients with acute myocardial infarction of anterior wall: randomized clinical trial: Impact of bone marrow stem cell intracoronary infusion on improvement of microcirculation. Eur Heart J 31: 691-702.

6. Hirsch A, Nijveldt R, van der Vleuten PA, Tijssen JG, van der Giessen WJ, et al. (2010) Intracoronary infusion of mononuclear cells from bone marrow or peripheral blood compared with standard therapy in patients after acute myocardial infarction treated by primary percutaneous coronary intervention: results of the randomized controlled HEBE trial. Eur Heart J.

7. Huang RC, Yao K, Zou YZ, Ge L, Qian JY, et al. (2006) [Long term follow-up on emergent intracoronary autologous bone marrow mononuclear cell transplantation for acute inferior-wall myocardial infarction]. Zhonghua Yi Xue Za Zhi 86: 1107-1110.

8. Huang L, Hou D, Thompson MA, Baysden SE, Shelley WC, et al. (2007) Acute myocardial infarction in swine rapidly and selectively releases highly proliferative endothelial colony forming cells (ECFCs) into circulation. Cell Transplant 16: 887-897.

9. Huikuri HV, Kervinen K, Niemela M, Ylitalo K, Saily M, et al. (2008) Effects of intracoronary injection of mononuclear bone marrow cells on left ventricular function, arrhythmia risk profile, and restenosis after thrombolytic therapy of acute myocardial infarction. Eur Heart J 29: 2723-2732.

10. Janssens S, Dubois C, Bogaert J, Theunissen K, Deroose C, et al. (2006) Autologous bone marrow-derived stem-cell transfer in patients with ST-segment elevation myocardial infarction: double-blind, randomised controlled trial. Lancet 367: 113-121.

11. Jin B, Yang Y, Shi H, Luo X, Li Y, et al. (2008) Autologous intracoronary mononuclear bone marrow cell transplantation for acute anterior myocardial infarction: Outcomes after 12-month follow-up. Journal of Clinical Rehabilitative Tissue Engineering Research 12: 2267-2271.

12. Kang HJ, Lee HY, Na SH, Chang SA, Park KW, et al. (2006) Differential effect of intracoronary infusion of mobilized peripheral blood stem cells by granulocyte colony-stimulating factor on left ventricular function and remodeling in patients with acute myocardial infarction versus old myocardial infarction: the MAGIC Cell-3-DES randomized, controlled trial. Circulation 114: I145-151.

13. Karpov RS, Popov SV, Markov VA, Suslova TE, Ryabov VV, et al. (2005) Autologous mononuclear bone marrow cells during reparative regeneratrion after acute myocardial infarction. Bull Exp Biol Med 140: 640-643.

14. Li ZQ, Zhang M, Jing YZ, Zhang WW, Liu Y, et al. (2007) The clinical study of autologous peripheral blood stem cell transplantation by intracoronary infusion in patients with acute myocardial infarction (AMI). Int J Cardiol 115: 52-56.

15. Lunde K, Solheim S, Aakhus S, Arnesen H, Abdelnoor M, et al. (2006) Intracoronary injection of mononuclear bone marrow cells in acute myocardial infarction. N Engl J Med 355: 1199-1209.

16. Meluzin J, Janousek S, Mayer J, Groch L, Hornacek I, et al. (2008) Three-, 6-, and 12-month results of autologous transplantation of mononuclear bone marrow cells in patients with acute myocardial infarction. Int J Cardiol 128: 185-192.

17. Meyer GP, Wollert KC, Lotz J, Steffens J, Lippolt P, et al. (2006) Intracoronary bone marrow cell transfer after myocardial infarction: eighteen months' follow-up data from the randomized, controlled BOOST (BOne marrOw transfer to enhance ST-elevation infarct regeneration) trial. Circulation 113: 1287-1294.

18. Nogueira FB, Silva SA, Haddad AF, Peixoto CM, Carvalho RM, et al. (2009) Systolic function of patients with myocardial infarction undergoing autologous bone marrow transplantation. Arq Bras Cardiol 93: 374-379, 367-372.

19. Penicka M, Horak J, Kobylka P, Pytlik R, Kozak T, et al. (2007) Intracoronary injection of autologous bone marrow-derived mononuclear cells in patients with large anterior acute myocardial infarction: a prematurely terminated randomized study. J Am Coll Cardiol 49: 2373-2374.

20. Piepoli MF, Vallisa D, Arbasi M, Cavanna L, Cerri L, et al. (2010) Bone marrow cell transplantation improves cardiac, autonomic, and functional indexes in acute anterior myocardial infarction patients (Cardiac Study). Eur J Heart Fail 12: 172-180.

21. Plewka M, Krzeminska-Pakula M, Lipiec P, Peruga JZ, Jezewski T, et al. (2009) Effect of intracoronary injection of mononuclear bone marrow stem cells on left ventricular function in patients with acute myocardial infarction. Am J Cardiol 104: 1336-1342.

22. Quyyumi AA, Waller EK, Murrow J, Esteves F, Galt J, et al. (2011) CD34(+) cell infusion after ST elevation myocardial infarction is associated with improved perfusion and is dose dependent. Am Heart J 161: 98-105.

23. Roncalli J, Mouquet F, Piot C, Trochu JN, Le Corvoisier P, et al. (2010) Intracoronary autologous mononucleated bone marrow cell infusion for acute myocardial infarction: results of the randomized multicenter BONAMI trial. Eur Heart J.

24. Ruan W, Pan CZ, Huang GQ, Li YL, Ge JB, et al. (2005) Assessment of left ventricular segmental function after autologous bone marrow stem cells transplantation in patients with acute myocardial infarction by tissue tracking and strain imaging. Chin Med J (Engl) 118: 1175-1181.

25. Schachinger V, Erbs S, Elsasser A, Haberbosch W, Hambrecht R, et al. (2006) Intracoronary bone marrow-derived progenitor cells in acute myocardial infarction. N Engl J Med 355: 1210-1221.

26. Suarez de Lezo J, Herrera CP, M., Romero M, Pavlovic D, Segura J, et al. (2007) Tratamiento regenerativo en pacientes con infarto agudo anterior revascularizado y funcion ventricular deprimida. Rev Esp Cardiol 60: 357-365.

27. Tendera M, Wojakowski W, Ruzyllo W, Chojnowska L, Kepka C, et al. (2009) Intracoronary infusion of bone marrow-derived selected CD34+CXCR4+ cells and non-selected mononuclear cells in patients with acute STEMI and reduced left ventricular ejection fraction: results of randomized, multicentre Myocardial Regeneration by Intracoronary Infusion of Selected Population of Stem Cells in Acute Myocardial Infarction (REGENT) Trial. Eur Heart J 30: 1313-1321.

28. Traverse JH, McKenna DH, Harvey K, Jorgenso BC, Olson RE, et al. (2010) Results of a phase 1, randomized, double-blind, placebo-controlled trial of bone marrow mononuclear stem cell administration in patients following ST-elevation myocardial infarction. Am Heart J 160: 428-434.

29. Wohrle J, Merkle N, Mailander V, Nusser T, Schauwecker P, et al. (2010) Results of intracoronary stem cell therapy after acute myocardial infarction. Am J Cardiol 105: 804-812.

30. Yao K, Huang RC, Ge L, Qian JY, Li YL, et al. (2006) [Observation on the safety: clinical trail on intracoronary autologous bone marrow mononuclear cells transplantation for acute myocardial infarction]. Zhonghua Xin Xue Guan Bing Za Zhi 34: 577-581.

31. Yao K, Huang R, Sun A, Qian J, Liu X, et al. (2009) Repeated autologous bone marrow mononuclear cell therapy in patients with large myocardial infarction. Eur J Heart Fail 11: 691-698.

32. You Q, Shen Z, Xiao M, Jiang X (2008) Influence of autologous bone marrow stem cell transplantation on short-term heart function in cardiac failure patients. Journal of Clinical Rehabilitative Tissue Engineering Research 12: 1467-1471.

33. Zhukova NS, Staroverov, II, Stukalova OV, Samoleinko LE, Romanov Iu A, et al. (2009) [An experience of the use of stem cells in the treatment of patients with myocardial infarction and low ejection fraction]. Kardiologiia 49: 19-24.

34. Roncalli J, Mouquet F, Piot C, Trochu JN, Le Corvoisier P, et al. (2011) Intracoronary autologous mononucleated bone marrow cell infusion for acute myocardial infarction: results of the randomized multicenter BONAMI trial. Eur Heart J 32: 1748-1757.

35. Hirsch A, Nijveldt R, van der Vleuten PA, Tijssen JG, van der Giessen WJ, et al. (2011) Intracoronary infusion of mononuclear cells from bone marrow or peripheral blood compared with standard therapy in patients after acute myocardial infarction treated by primary percutaneous coronary intervention: results of the randomized controlled HEBE trial. Eur Heart J 32: 1736-1747.
